# ﻿Complete mitochondrial genomes of the slugs *Deroceraslaeve* (Agriolimacidae) and *Ambigolimaxvalentianus* (Limacidae) provide insights into the phylogeny of Stylommatophora (Mollusca, Gastropoda)

**DOI:** 10.3897/zookeys.1173.102786

**Published:** 2023-07-31

**Authors:** Te Zhao, Nan Song, Xingyu Lin, Yang Zhang

**Affiliations:** 1 College of Plant Protection, Henan Agricultural University, Zhengzhou 450002, China Henan Agricultural University Zhengzhou China

**Keywords:** Gene rearrangement, Limacoidea, mitogenome, next generation sequencing, phylogeny

## Abstract

In this study, we sequenced two complete mitogenomes from *Deroceraslaeve* and *Ambigolimaxvalentianus*. The mitogenome of *Ambigolimaxvalentianus* represented the first such data from the family Limacidae. The lengths of the mitogenomes of *Deroceraslaeve* and *Ambigolimaxvalentianus* were 14,773 bp and 15,195 bp, respectively. The entire set of 37 mitochondrial genes were identified for both mitogenomes. Compared with the mitogenome of *Achatinafulica*, the *trnP*_*trnA* tRNA cluster was rearranged in both *Deroceraslaeve* and *Ambigolimaxvalentianus*. The secondary structures of tRNA and rRNA genes for the two species were predicted. Phylogenetic analyses based on amino acid sequences supported (1) monophyly of Stylommatophora, (2) division of Stylommatophora into the ‘achatinoid’ clade (i.e., the suborder Achatinina) and the ‘non-achatinoid’ clade (i.e., the suborder Helicina), (3) placement of the Orthurethra in the ‘non-achatinoid’ clade, and (4) monophyly of each of the superfamilies Helicoidea, Urocoptoidea, Succineoidea, Arionoidea, Pupilloidea and Limacoidea. The exemplars of Helicidae, Philomycidae and Achatinellidae displayed many more mitochondrial gene rearrangements than other species of Stylommatophora.

## ﻿Introduction

Typically, the metazoan mitochondrial genome (mitogenome) is a closed-circular and small (15–20 kb) genome encoding 13 protein-coding genes (PCGs), 22 transfer RNAs (tRNAs) and two ribosomal RNAs (rRNAs) (Boore 1999). Mitogenomes have been widely used to resolve phylogenetic relationships within molluscs (e.g., He et al. 2016; Minton et al. 2016; Xie et al. 2019; Guzmán et al. 2021). With the development of new sequencing technologies and the significantly decreased cost of next generation sequencing, the numbers of available mitogenomes has increased rapidly. Molluscs are the second largest phylum next to Arthropoda, with about 52,500 extant species (Ponder et al. 2020). However, relatively few mitogenomes are available for this group. Within Mollusca, Stylommatophora includes the vast majority of terrestrial snails and slugs. As of December 2022, only 58 stylommatophoran mitogenomes were available in GenBank.

The Stylommatophora is the largest group within the pulmonate gastropods, containing 20,000 ± 1500 species (Rosenberg et al. 2022). Based on the structure of the excretory system Pilsbry (1900) divided the Stylommatophora in three infraorders: Orthurethra, Heterurethra and Sigmurethra. Baker (1955) adapted this system by recognizing a fourth major group, viz. Mesurethra. Of these four taxa, only Orthurethra is still widely accepted as a natural group. Deep-level relationships within Stylommatophora have been controversial. Nordsieck (1992) suggested dividing Stylommatophora into two subclades, Orthurethra and Sigmurethra. The first comprehensive molecular study of stylommatophoran relationships was undertaken by Wade et al. (2001), who recognized an ‘achatinoid’ clade and a ‘non-achatinoid’ clade. This hypothesis was subsequently supported by Wade et al. (2006). Bouchet et al. (2005) divided the Stylommatophora into three clades, Elasmognatha, Orthurethra and Sigmurethra, based a combined analysis of morphological and molecular data. Elasmognatha contains Succineoidea and Athoracophoroidea. Orthurethra contains Partuloidea, Achatinelloidea, Cochlicopoidea, Pupilloidea and Enoidea. Sigmurethra was suggested to be an informal group, which contains Clausilioidea, Orthalicoidea, Achatinoidea, Aillyoidea, Testacelloidea, Papillodermatoidea, Streptaxoidea, Rhytidoidea, Acavoidea, Plectopyloidea, Punctoidea and Sagdoidea. Bouchet et al. (2017) recognized Stylommatophora as an order and divided it into three suborders, Achatinina, Helicina and Scolodontina. Achatinina comprises Achatinoidea and Streptaxoidea. Helicina includes Coelociontoidea, Papillodermatoidea, Plectopyloidea, Punctoidea, Testacelloidea and Urocoptoidea. Scolodontina contains the single family Scolodontidae. Saadi and Wade (2019) further refined this hypothesis by recognizing Scolodontidae as sister to all other stylommatophoran groups comprising the ‘achatinoid’ and ‘non-achatinoid’ clades. Scolodontidae corresponded to the suborder Scolodontina proposed by Bouchet et al. (2017), while the ‘achatinoid’ clade corresponded to the suborder Achatinina and the ‘non-achatinoid’ clade corresponded to the suborder Helicina.

Limacoidea is a superfamily of Stylommatophora that is subdivided into four families: Agriolimacidae, Limacidae, Boettgerillidae and Vitrinidae (Hausdorf 1998; Bouchet et al. 2017). The slug *Ambigolimaxvalentianus* is an invasive species in North and South America, Africa and Asia (Robinson 1999). Several studies have proved the usefulness of mitogenome data to resolve phylogenetic relationships in Stylommatophora (Minton et al. 2016; Xie et al. 2019; Guzmán et al. 2021). In this study, we applied next generation sequencing to obtain the complete mitogenomes of *Derocerasleave* (O. F. Müller, 1774) and *Ambigolimaxvalentianus* (A. Férussac, 1821). The mitogenome of *Deroceraslaeve* represented the second for Agriolimacidae and that of *Ambigolimaxvalentianus* was the first for Limacidae. This contribution aims at characterizing these two new mitogenomes and using them for a phylogenetic analysis of Stylommatophora.

## ﻿Material and methods

### ﻿Specimens and DNA extraction

Specimens of *Deroceraslaeve* and *Ambigolimaxvalentianus* were collected from Zunyi, Guizhou Province, China, in July, 2020. They were identified by checking their adult morphological characters and blasting the mitochondrial *cox1* gene sequences in the BOLD system. The voucher specimens were deposited at the Henan Agricultural University, Zhengzhou, China, under the accession numbers MT-Zy20200701 and MT-Zy20200702. The specimens were preserved in absolute ethanol, and stored at -80 °C until DNA extraction. Total genomic DNA of the individual specimen was extracted with the TIANamp Genomic DNA Kit (TIANGEN BIOTECH CO., LTD), following the manufacturer’s protocol.

### ﻿Genome sequencing, assembly and annotation

Genome sequencing was performed on an Illumina HiSeq2500 platform, with a strategy of 150 paired-end sequencing. Library generation for the Illumina Hiseq sequencing was carried out using the Illumina TruSeqTM DNA Sample Prep Kit (Illumina, San Diego, CA, USA), with 350 bp insert size. NGS QC Toolkit v.2.3.3 (Patel and Jain 2012) was used to check the quality of the data. Adapters, ploy-N, and low-quality reads were removed from raw data. About 3 Gb clean data obtained by NGS for each species were used to assemble the mitochondrial scaffold.

GetOrganelle v.1.7.5.2 (Jin et al. 2020) was used for mitogenome assembly. The GetOrganelle animal database (-F animal_mt) was applied to identify, filter, and assemble target-associated reads. The new mitogenomes were annotated with the MITOS webserver (Bernt et al. 2013) (http://mitos2.bioinf.uni-leipzig.de/index.py). The gene boundaries of protein-coding genes were refined by alignment against mitochondrial gene sequences of closely related species. tRNA genes were identified using MITOS (Bernt et al. 2013) and ARWEN (Laslett and Canbäck 2008), and the secondary structures were redrawn in Adobe Illustrator CC 2019. The secondary structures of rRNA genes were predicted with reference to *Omalonyxunguis* (Guzmán et al. 2021). The mitogenome structure images were generated using mtviz (http://pacosy.informatik.uni-leipzig.de/mtviz). The annotated mitogenome sequences were submitted to GenBank under the accession numbers of OQ198714 (*Deroceraslaeve*) and OQ198715 (*Ambigolimaxvalentianus*).

### ﻿Characterization of the new mitogenomes

Pairwise comparisons of gene order with the gene order of *Achatinafulica* (He et al. 2016; Yang et al. 2016; Xie et al. 2019) and assessment of rearrangement events were performed using CREx (http://pacosy.informatik.uni-leipzig.de/crex/form) (Bernt et al. 2007). The nucleotide compositions of the mitogenome sequences were calculated with MEGA 11 (Kumar et al. 2018). AT and GC-skew values were obtained using the following formulas: AT-skew = (A-T)/(A+T) and GC-skew = (G-C)/(G+C) (Perna and Kocher 1995).

### ﻿Sequence alignment

Protein-coding genes were aligned individually using MUSCLE as implemented in MEGA 11 (with default settings) (Kumar et al. 2018). Protein-coding genes were translated into amino acid sequences using the invertebrate mitochondrial genetic code. The alignments of genes were concatenated with FASconCAT-G_v.1.04 (Kück and Longo 2014) to create the amino acid dataset PCG_aa.

### ﻿Phylogenetic analysis

A total of 68 mollusk mitogenome sequences were used in the phylogenetic analyses, of which 59 species were included as ingroup to represent Stylommatophora. Four species from Systellommatophora, two species from Ellobiida and three species from Hygrophila were selected as outgroups (Suppl. material 1). Phylogenetic analyses were performed based on the amino acid dataset mentioned above, under maximum likelihood (ML) and Bayesian inference (BI) criteria. ML analysis was performed with IQ-TREE v.1.6.10 (Nguyen et al. 2015). The data was partitioned by gene types. The best-fitting substitution models for partitions were chosen using ModelFinder (Kalyaanamoorthy et al. 2017) implemented in IQ-TREE. Branch support (BS) values were calculated using ultrafast bootstrap with 10,000 replicates. BI analysis was conducted using MrBayes v.3.2.7 (Ronquist et al. 2012). Two runs with four chains each were performed. The initial number of generations for each run was set to 10 million. Sampling was done every 1000 generations. The Average Standard Deviation of Split Frequencies (ASDSF) were monitored using Tracer v.1.7 (Rambaut et al. 2018). After reaching convergence (ASDSF < 0.01), the tree and branch length information were summarized using the *sumt* command, discarding the first 25% as burn-in. The consensus tree was yielded, and posterior probability (PP) values were used to assess branch support.

## ﻿Results

### ﻿Characteristics of the new mitogenomes

**Figure 1.**
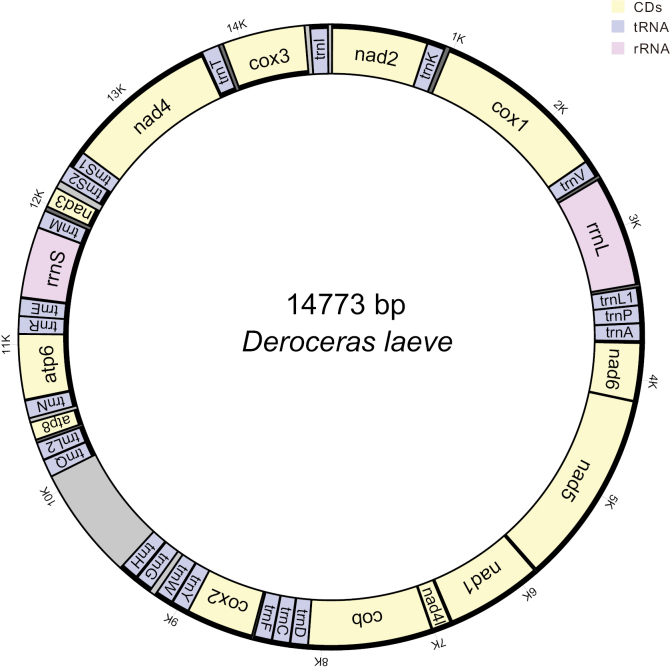
Gene order and gene content of the mitogenome of *Deroceraslaeve*. The abbreviation of genes follows MITOS. Starting from *trnI* and ending at *cox3*, count clockwise. The outer numbers indicate the positions of each section.

**Figure 2.**
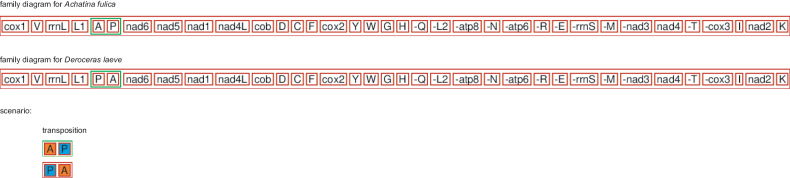
The rearrangement event assessed from the CREx analysis for the mitogenome of *Deroceraslaeve*.

The entire mitochondrial genomes of *Deroceraslaeve* and *Ambigolimaxvalentianus* were 14,773 base pairs (bp) and 15,195 bp long, respectively. They contained the entire set of 37 genes usually present in the animal mitogenomes and had identical gene orders (Fig. 1). Compared with the gene order of *Achatinafulica* (He et al. 2016; Yang et al. 2016; Xie et al. 2019), the *trnP*_*trnA* cluster was rearranged in both *Deroceraslaeve* and *Ambigolimaxvalentianus*. The CREx analysis showed that the rearranged gene order of *Deroceraslaeve* and *Ambigolimaxvalentianus* has evolved as the result of transposition (Fig. 2). The nucleotide compositions of both mitogenomes were heavily biased towards A and T. The overall A+T content of *Deroceraslaeve* was 73.0%, while the A+T content of *Ambigolimaxvalentianus* was 71.4%. Both mitogenomes had the negative AT-skew values (-0.115 for *Deroceraslaeve* and -0.096 for *Ambigolimaxvalentianus*) and the positive GC-skew values (0.181 for *Deroceraslaeve* and 0.113 for *Ambigolimaxvalentianus*) in the major strand. This indicated the occurrence of more T than A and more G than C.

**Figure 3.**
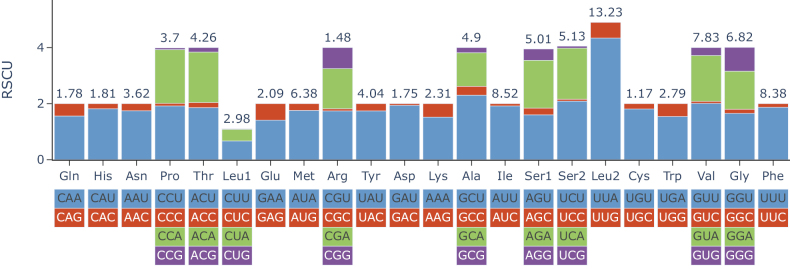
Codon usage of the 13 mitochondrial protein-coding genes of *Deroceraslaeve*. RSCU: relative synonymous codon usage.

For the protein-coding genes, ATG (five for *Deroceraslaeve* and four for *Ambigolimaxvalentianus*), ATT (six for *Deroceraslaeve* and three for *Ambigolimaxvalentianus*) and ATA (one for *Ambigolimaxvalentianus*) were used as the start codons. For the *cox2* gene and *atp8* gene of *Ambigolimaxvalentianus*, GTG was the start codon. For the *cox1* gene and *cob* gene of both *Ambigolimaxvalentianus* and *Deroceraslaeve*, TTG was the start codon. All protein-coding genes terminated with the stop codon TAA or TAG, except for *nad3* and *nad4L* of *Ambigolimaxvalentianus*, which had the incomplete stop codon T. For both species, Leu, Ile, Phe and Val were the most frequently used amino acids. Relative synonymous codon usage (RSCU) for 13 protein-coding genes of *Deroceraslaeve* is shown in Fig. 3. *Ambigolimaxvalentianus* (Suppl. material 2) had similar RSCU values to *Deroceraslaeve*.

**Figure 4.**
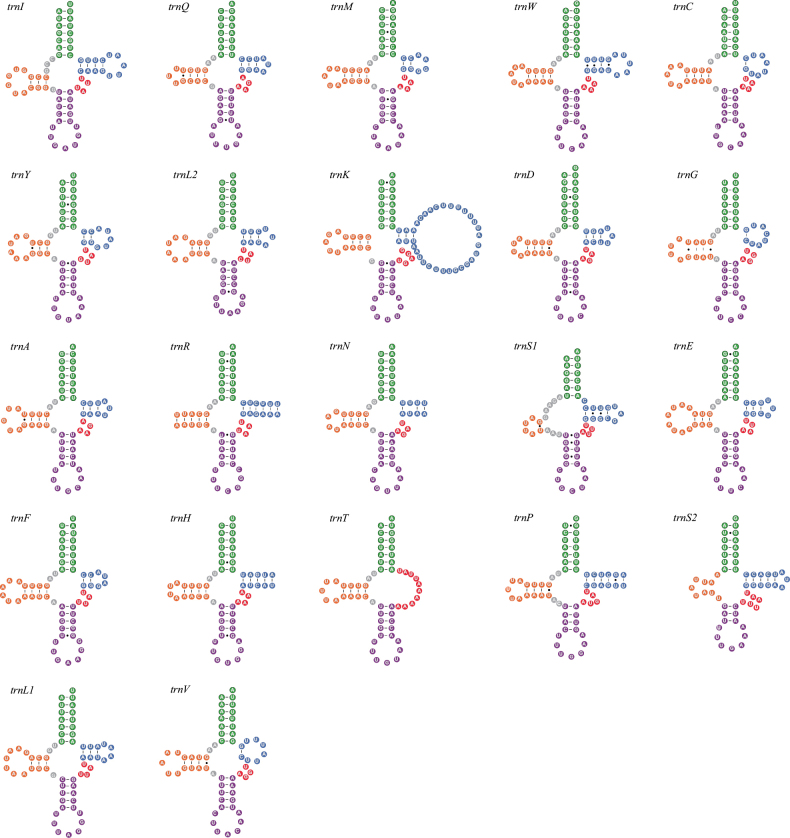
The secondary structures of tRNA genes inferred for the mitogenome of *Deroceraslaeve*.

**Figure 5.**
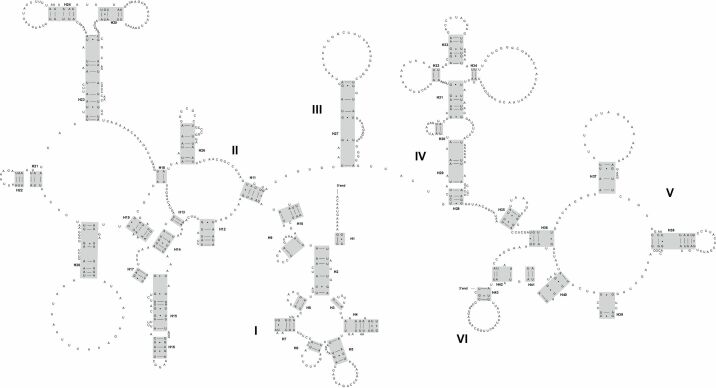
The secondary structure of rrnL inferred for *Deroceraslaeve*.

**Figure 6.**
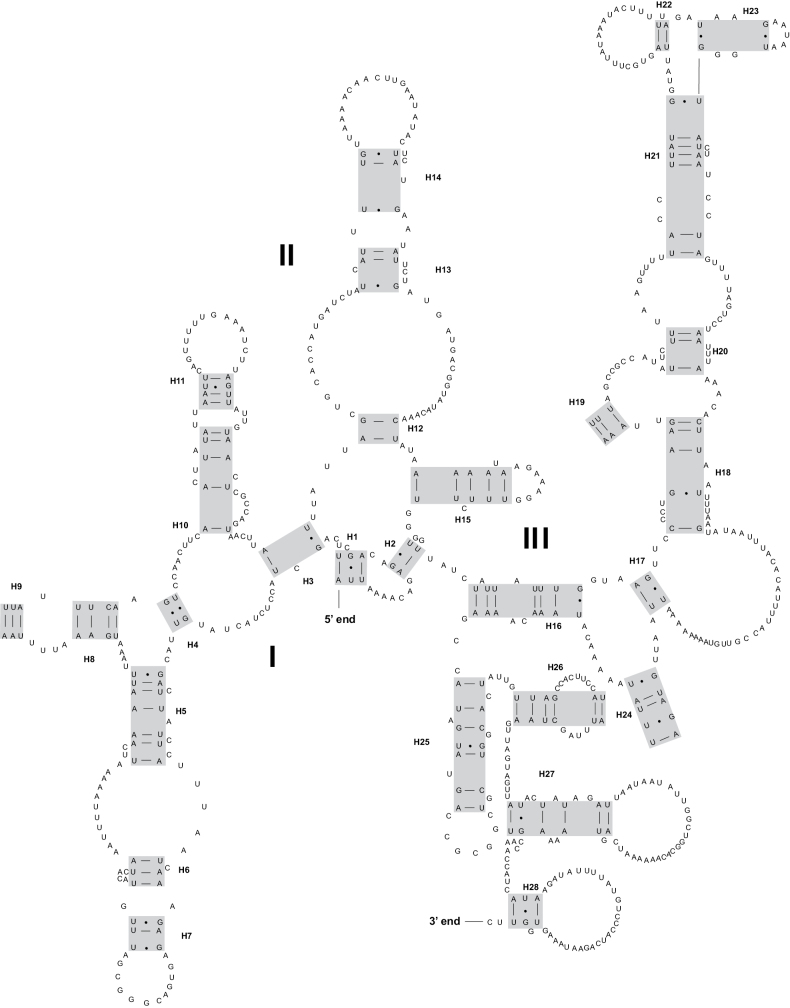
The secondary structure of *rrnS* inferred for *Deroceraslaeve*.

All 22 tRNA genes were identified by both MITOS and ARWEN, and their lengths ranged from 61 to 68 bp. Most tRNA genes of both species can be folded into the classic clover-leaf structure (Fig. 4 and Suppl. material 3). The *trnT* gene of *Deroceraslaeve* had an unusual TΨC loop. The *trnK* gene and *trnS1* gene of *Ambigolimaxvalentianus* had an incomplete DHU arm. The position of the *rrnL* gene was located between *trnV* and *trnL1*, while the *rrnS* gene was found between *trnE* and *trnM*. In *Deroceraslaeve*, *rrnL* had a length of 1052 bp with an A + T content of 74.4%, whereas *rrnS* had a length of 697 bp with an A + T content of 71.5%. In *Ambigolimaxvalentianus*, *rrnL* was 1091 bp long with an A + T content of 75.9%, while *rrnS* was 711 bp long with an A + T content of 72.7%. The secondary structures for *rrnL* and *rrnS* of *Deroceraslaeve* and *Ambigolimaxvalentianus* are presented in Figs 5, 6 and Suppl. materials 4, 5, respectively. The *rrnL* molecule of both species contained six domains (labeled I–VI) comprising 43 helices. The *rrnS* molecule consisted of three domains (labeled I–III) and 28 helices.

### ﻿Phylogenetic inference

**Figure 7.**
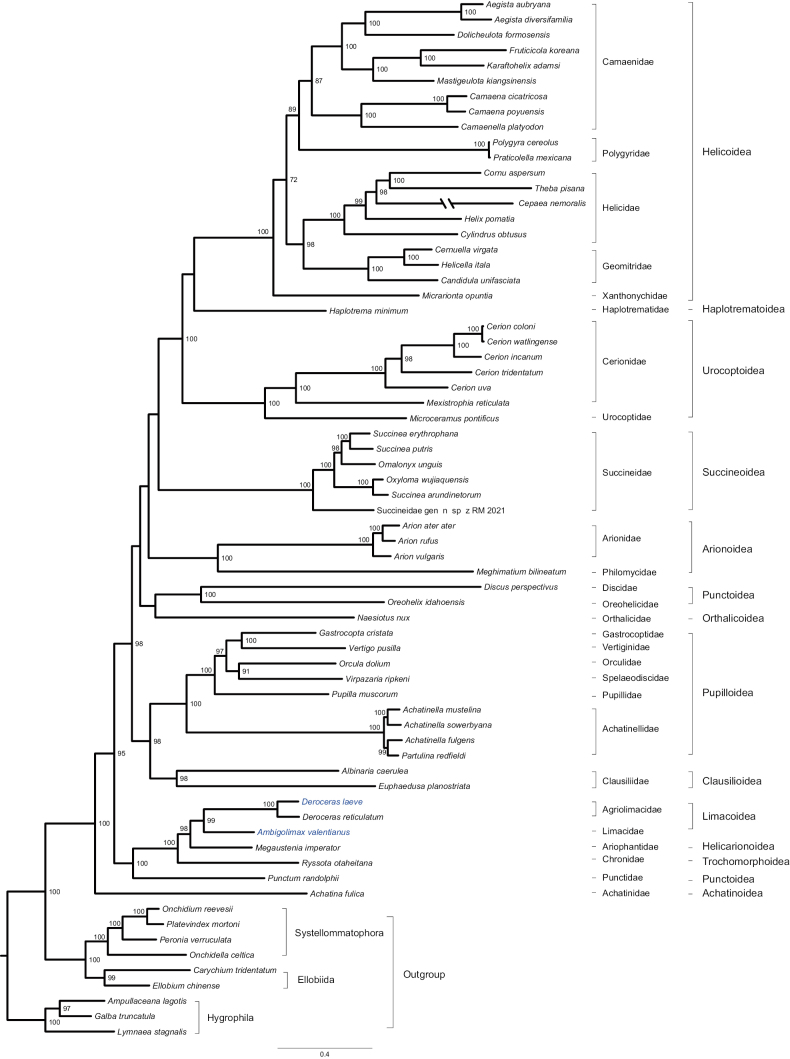
ML phylogenetic tree inferred with IQ-TREE using amino acid sequences of 13 PCGs. Numbers at the nodes are ultrafast bootstrap values (BS > 70). Blue indicates the newly sequenced species. The branch of *Cepaeanemoralis* is depicted as half of its original branch length. Scale bar represents substitutions/site.

**Figure 8.**
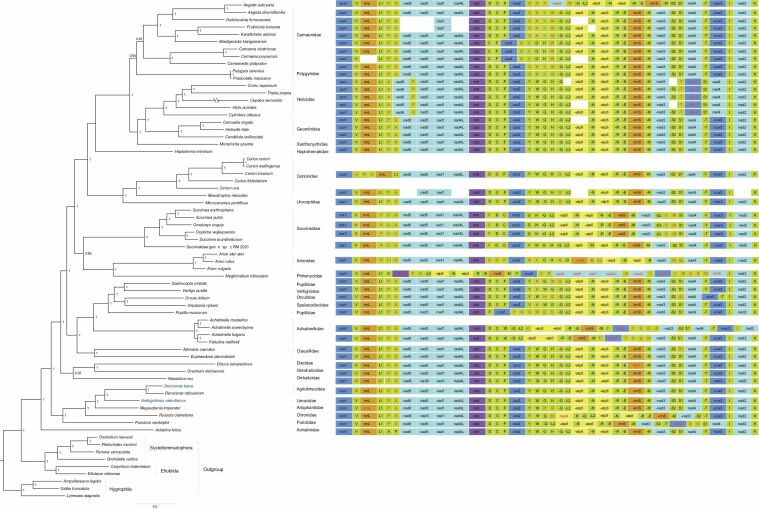
Bayesian phylogenetic tree inferred in MrBayes using amino acid sequences of 13 PCGs (left), and gene order comparisons for the stylommatophoran mitogenomes (right). Numbers at the nodes in the tree are Bayesian posterior probabilities (PP > 0.9). Blue indicates the newly sequenced species. The branch of *Cepaeanemoralis* is depicted as half of its original branch length. Scale bar represents substitutions/site. In the mitogenome structure maps, gene rearrangements are highlighted by red.

ML and BI produced similar tree topologies. The monophyly of Stylommatophora was supported under both analyses (Figs 7, 8). Achatinoidea represented by *Achatinafulica* was consistently resolved as the sister group of all other Stylommatophora (BS = 100, PP = 1.0). This lineage corresponded to the suborder Achatinina. The remaining stylommatophorans formed the ‘non-achatinoid’ clade which corresponded to the suborder Helicina. In Helicina, all superfamilies with more than two representatives were well supported, with the exception of Punctoidea. All families with multiple representatives were supported as monophyletic. The newly sequenced *Deroceraslaeve* was sister to *Derocerasreticulatum*, and both together formed the sister group of *Ambigolimaxvalentianus*. The three species form a monophyletic Limacoidea clade.

The major differences between the ML and BI analyses were the positions of Xanthonychidae and the clade Orthalicoidea + Punctoidea. The ML analysis placed Xanthonychidae as sister to all other Helicoidea. Whereas, the BI analysis recovered Xanthonychidae as the sister group of a clade Geomitridae + Helicidae. The ML analysis placed Orthalicoidea + Punctoidea between a clade comprising (Clausilioidea + Pupilloidea) and Arionoidea comprising (Philomycidae + Arionidae). But this arrangement received no statistical support. The BI analysis recovered Orthalicoidea + Punctoidea between the clade (Clausilioidea + Pupilloidea) and a clade including Limacoidea, Helicarionoidea, Trochomorphoidea and Punctidae.

## ﻿Discussion

### ﻿Mitochondrial gene rearrangement

Large-scale changes in genomes are considered to be rare events (Rokas and Holland 2000). The gene set remains constant across bilaterian animals, mitochondrial gene rearrangements appear to be unusual, and gene order is selectively neutral (Boore et al. 1995; Dowton et al. 2002; Dowton et al. 2009; Cameron 2014). In a certain animal group, for example the hexapods, most species share an identical mitogenome organization (Dowton et al. 2002; Cameron 2014). However, Stylommatophora mitogenomes have experienced many more mitochondrial gene rearrangements than other groups (Minton et al. 2016; Xie et al. 2019; Guzmán et al. 2021). In this study, two new mitogenomes from *Deroceraslaeve* and *Ambigolimaxvalentianus* had a tRNA gene rearrangement associated with the *trnP*_*trnA* tRNA cluster. We compared the gene order for all included mitogenomes and found that all the Helicina species have gene rearrangements compared to *Achatinafulica*. The exemplars of Helicidae, Philomycidae and Achatinellidae displayed many more mitochondrial gene rearrangements than others (Fig. 8).

### ﻿Phylogeny of Stylommatophora

Within Stylommatophora, the division of the order into Achatinina and Helicina is well accepted (Wade et al. 2001; Wade et al. 2006). Recently, some authors added Scolodontidae to the phylogenetic analysis (Ramírez et al. 2012; Bouchet et al. 2017; Saadi and Wade 2019). Currently, no mitogenomes of Scolodontidae have been published, so the phylogenetic position of Scolodontidae could not be assessed in this analysis. Our analyses consistently supported the division of Stylommatophora into two principal clades, Achatinina and Helicina. This result is consistent with the previous mitogenome analyses (Xie et al. 2019; Guzmán et al. 2021).

The present mitogenome data recovered Clausilioidea as a sister group of the orthurethran clade. This result contrasted with Wade et al. (2006), who recovered Orthurethra to be close to a clade comprising Arionoidea and Limacoidea. In a prior mitogenome analysis, a sister group relationship between Succineoidea and Arionoidea was supported (Xie et al. 2019). In this study, this pattern was supported by the BI analysis (PP = 0.96) based on expanded taxon sampling of mitogenomes. In our analyses, only one species of *Haplotremaminimum* (Ancey, 1888) representing Haplotrematoidea was included due to mitogenome data availability. Both the ML and BI analyses placed Haplotrematoidea as sister to Helicoidea. However, this relationship had low support values. In future researches, larger taxon samples are needed to identify the phylogenetic placement of Haplotrematoidea.

The superfamily Punctoidea included the families Punctidae, Charopidae, Cystopeltidae, Discidae (“Endodontidae”), Helicodiscidae, Oopeltidae and Oreohelicidae (Bouchet et al. 2017). In this study, we included three species of Punctoidea in the phylogenetic analyses, which respectively represented Discidae [*Discusperspectivus* (Megerle von Mühlfeld, 1816)], Oreohelicidae [*Oreohelixidahoensis* (Newcomb, 1866)] and Punctidae [*Punctumrandolphii* (Dall, 1895)]. *Discusperspectivus* and *O.idahoensis* were significantly supported to be a sister group. *Punctumrandolphii* was placed separately, and clustered with a clade including Trochomorphoidea, Helicarionoidea and Limacoidea. Taxon sampling of Punctoidea was very limited in our analysis. The monophyly of Punctoidea needs to be further tested by additional species in future studies.

Previous analyses based on the multiple gene fragments have demonstrated the monophyly of Helicoidea, which comprised the families Helicidae, Bradybaenidae, Xanthonychidae, Hygromiidae, Camaenidae, Polygyridae and Sagdidae (Wade et al. 2006). Our results strongly supported a monophyletic Helicoidea (BS = 100, PP = 1.0). In addition, the superfamilies Urocoptoidea, Succineoidea, Arionoidea, Pupilloidea and Limacoidea were supported to be monophyletic groups. These patterns are congruent with the current taxonomy of land snails (Bouchet et al. 2017).

For the taxon sampling of outgroups, we included four exemplars from Systellommatophora and two from Ellobiida. The monophyly of Systellommatophora and Ellobiida were consistently supported in both ML and BI analyses. Moreover, Systellommatophora and Ellobiida formed a sister-group relationship (BS = 100, PP = 1). Amphipulmonata Schrödl, 2014 was established as a clade containing Systellommatophora and Ellobiida (Bouchet et al. 2017). Amphipulmonata (comprising Ellobioidea and Systellommatophora) was supported in the previous molecular phylogenetic analysis (Dayrat et al. 2011). Our results confirmed the hypothesis of Amphipulmonata.

## ﻿Additional information

### Data availability

The mitogenome sequences newly generated in this study were deposited in GenBank, with the accession numbers of OQ198714–OQ198715. The dataset used for phylogenetic analyses can be obtained from the corresponding author upon request.

## Supplementary materials

Supplementary material 1 Taxa included in this study, including GenBank Accession numbers and literature references Data type table (docx. file) https://binary.pensoft.net/file/886302This dataset is made available under the Open Database License (http://opendatacommons.org/licenses/odbl/1.0/). The Open Database License (ODbL) is a license agreement intended to allow users to freely share, modify, and use this Dataset while maintaining this same freedom for others, provided that the original source and author(s) are credited.Te Zhao, Nan Song, Xingyu Lin, Yang Zhang

Supplementary material 2 Relative synonymous codon usage (RSCU) for 13 protein-coding genes of *Ambigolimaxvalentianus* Data type figure (PDF file) https://binary.pensoft.net/file/886303This dataset is made available under the Open Database License (http://opendatacommons.org/licenses/odbl/1.0/). The Open Database License (ODbL) is a license agreement intended to allow users to freely share, modify, and use this Dataset while maintaining this same freedom for others, provided that the original source and author(s) are credited.Te Zhao, Nan Song, Xingyu Lin, Yang Zhang

Supplementary material 3 The secondary structures of tRNA genes inferred for the mitogenome of *Ambigolimaxvalentianus* Data type figure (PDF file) https://binary.pensoft.net/file/886304This dataset is made available under the Open Database License (http://opendatacommons.org/licenses/odbl/1.0/). The Open Database License (ODbL) is a license agreement intended to allow users to freely share, modify, and use this Dataset while maintaining this same freedom for others, provided that the original source and author(s) are credited.Te Zhao, Nan Song, Xingyu Lin, Yang Zhang

Supplementary material 4 The secondary structure of *rrnL* inferred for *Ambigolimaxvalentianus* Data type figure (PDF file) https://binary.pensoft.net/file/886305This dataset is made available under the Open Database License (http://opendatacommons.org/licenses/odbl/1.0/). The Open Database License (ODbL) is a license agreement intended to allow users to freely share, modify, and use this Dataset while maintaining this same freedom for others, provided that the original source and author(s) are credited.Te Zhao, Nan Song, Xingyu Lin, Yang Zhang

Supplementary material 5 The secondary structure of *rrnS* inferred for *Ambigolimaxvalentianus* Data type figure (PDF file) https://binary.pensoft.net/file/886306This dataset is made available under the Open Database License (http://opendatacommons.org/licenses/odbl/1.0/). The Open Database License (ODbL) is a license agreement intended to allow users to freely share, modify, and use this Dataset while maintaining this same freedom for others, provided that the original source and author(s) are credited.Te Zhao, Nan Song, Xingyu Lin, Yang Zhang
