# Supplementary material for: ﻿Complete mitochondrial genomes of the slugs Deroceraslaeve (Agriolimacidae) and Ambigolimaxvalentianus (Limacidae) provide insights into the phylogeny of Stylommatophora (Mollusca, Gastropoda)
Source: Zookeys. 2023 Jul 31;1173:43–59. doi: 10.3897/zookeys.1173.102786 (PMC10407649; doi:10.3897/zookeys.1173.102786)
Supplement: Supplementary material 1 — Taxa included in this study, including GenBank Accession numbers and literature references [file zookeys-1173-043_article-102786__-s001.docx]

| **Table S1** Taxa included in this study, including GenBank Accession numbers and literature references. | | | | | | |
| --- | --- | --- | --- | --- | --- | --- |
| **Order** | **Suborder** | **Superfamily** | **Family** | **Species** | **Accession number** | **References** |
| Stylommatophora | Helicina | Achatinoidea | Achatinidae | *Achatina fulica* | KJ744205 | He et al. 2016 |
| Stylommatophora | Helicina | Arionoidea | Arionidae | *Arion ater ater* | MW927710 | Unpublished |
| Stylommatophora | Helicina | Arionoidea | Arionidae | *Arion rufus* | KT626607 | Romero et al. 2016 |
| Stylommatophora | Helicina | Arionoidea | Arionidae | *Arion vulgaris* | MN607980 | Doğan et al. 2020 |
| Stylommatophora | Helicina | Arionoidea | Philomycidae | *Meghimatium bilineatum* | KP398563 | Unpublished |
| Stylommatophora | Helicina | Camaenoidea | Camaenidae | *Aegista aubryana* | KT192071 | Yang et al. 2016 |
| Stylommatophora | Helicina | Camaenoidea | Camaenidae | *Aegista diversifamilia* | KR002567 | Huang et al. 2016 |
| Stylommatophora | Helicina | Camaenoidea | Camaenidae | *Camaena cicatricosa* | KM365408 | Wang et al. 2014 |
| Stylommatophora | Helicina | Camaenoidea | Camaenidae | *Camaena poyuensis* | KT001074 | Lin et al. 2016 |
| Stylommatophora | Helicina | Camaenoidea | Camaenidae | *Camaenella platyodon* | MH362759 | Unpublished |
| Stylommatophora | Helicina | Camaenoidea | Camaenidae | *Dolicheulota formosensis* | KR338956 | Huang et al. 2016 |
| Stylommatophora | Helicina | Camaenoidea | Camaenidae | *Fruticicola koreana* | KU237291 | Unpublished |
| Stylommatophora | Helicina | Camaenoidea | Camaenidae | *Karaftohelix adamsi* | KY230382 | Unpublished |
| Stylommatophora | Helicina | Camaenoidea | Camaenidae | *Mastigeulota kiangsinensis* | KM083123 | Deng et al. 2016 |
| Stylommatophora | Helicina | Clausilioidea | Clausiliidae | *Albinaria caerulea* | NC_001761 | Hatzoglou et al. 1995 |
| Stylommatophora | Helicina | Clausilioidea | Clausiliidae | *Euphaedusa planostriata* | MW118059 | Unpublished |
| Stylommatophora | Helicina | Haplotrematoidea | Haplotrematidae | *Haplotrema minimum* | MT501508 | Unpublished |
| Stylommatophora | Helicina | Helicarionoidea | Ariophantidae | *Megaustenia imperator imperator* | OP171944 | Unpublished |
| Stylommatophora | Helicina | Helicoidea | Geomitridae | *Candidula unifasciata unifasciata* | MN747044 | Unpublished |
| Stylommatophora | Helicina | Helicoidea | Geomitridae | *Cernuella virgata* | KR736333 | Lin et al. 2016 |
| Stylommatophora | Helicina | Helicoidea | Geomitridae | *Helicella itala* | KT696546 | Romero et al. 2016 |
| Stylommatophora | Helicina | Helicoidea | Helicidae | *Cepaea nemoralis* | CMU23045 | Terrett et al. 1996 |
| Stylommatophora | Helicina | Helicoidea | Helicidae | *Cornu aspersum* | JQ417194 | Gaitán-Espitia et al. 2013 |
| Stylommatophora | Helicina | Helicoidea | Helicidae | *Cylindrus obtusus* | JN107636 | Groenenberg et al. 2012 |
| Stylommatophora | Helicina | Helicoidea | Helicidae | *Helix pomatia* | MK347426 | Petrusek and Rovatsos 2019 |
| Stylommatophora | Helicina | Helicoidea | Helicidae | *Theba pisana* | MH362760 | Wang et al. 2018 |
| Stylommatophora | Helicina | Helicoidea | Xanthonychidae | *Micrarionta opuntia* | MT527720 | Unpublished |
| Stylommatophora | Helicina | Limacoidea | Agriolimacidae | ***Deroceras laeve*** | OQ198714 | This study |
| Stylommatophora | Helicina | Limacoidea | Agriolimacidae | *Deroceras reticulatum* | KY765589 | Ahn et al. 2017 |
| Stylommatophora | Helicina | Limacoidea | Limacidae | ***Ambigolimax valentianus*** | OQ198715 | This study |
| Stylommatophora | Helicina | Orthalicoidea | Orthalicidae | *Naesiotus nux* | KT821554 | Hunter et al. 2016 |
| Stylommatophora | Helicina | Polygyroidea | Polygyridae | *Polygyra cereolus* | KX278421 | Minton et al. 2016 |
| Stylommatophora | Helicina | Polygyroidea | Polygyridae | *Praticolella mexicana* | KX240084 | Minton et al. 2016 |
| Stylommatophora | Helicina | Punctoidea | Discidae | *Discus perspectivus* | ON920776 | Unpublished |
| Stylommatophora | Helicina | Punctoidea | Oreohelicidae | *Oreohelix idahoensis* | MK290736 | Unpublished |
| Stylommatophora | Helicina | Punctoidea | Punctidae | *Punctum randolphii* | MT361346 | Unpublished |
| Stylommatophora | Helicina | Pupilloidea | Spelaeodiscidae | *Virpazaria ripkeni* | NC_063706 | Unpublished |
| Stylommatophora | Helicina | Succineoidea | Succineidae | *Omalonyx unguis* | MT449229 | Guzmán et al. 2021 |
| Stylommatophora | Helicina | Succineoidea | Succineidae | *Oxyloma wujiaquensis* | MT670402 | Unpublished |
| Stylommatophora | Helicina | Succineoidea | Succineidae | *Succinea arundinetorum* | OP289102 | Unpublished |
| Stylommatophora | Helicina | Succineoidea | Succineidae | *Succinea erythrophana* | ON533899 | Unpublished |
| Stylommatophora | Helicina | Succineoidea | Succineidae | *Succinea putris* | NC_016190 | White et al. 2011 |
| Stylommatophora | Helicina | Succineoidea | Succineidae | Succineidae gen. n. sp. z RM-2021 | OL681899 | Unpublished |
| Stylommatophora | Helicina | Trochomorphoidea | Chronidae | *Ryssota otaheitana* | MK716255 | Damatac et al. 2019 |
| Stylommatophora | Helicina | Urocoptoidea | Cerionidae | *Cerion coloni* | MN896024 | Unpublished |
| Stylommatophora | Helicina | Urocoptoidea | Cerionidae | *Cerion incanum* | KM365085 | Gonzalez et al. 2016 |
| Stylommatophora | Helicina | Urocoptoidea | Cerionidae | *Cerion tridentatum costellata* | KY249249 | Harasewych et al. 2011 |
| Stylommatophora | Helicina | Urocoptoidea | Cerionidae | *Cerion uva* | KY124261 | Harasewych et al. 2017 |
| Stylommatophora | Helicina | Urocoptoidea | Cerionidae | *Cerion watlingense* | MN904501 | Unpublished |
| Stylommatophora | Helicina | Urocoptoidea | Cerionidae | *Mexistrophia reticulata* | KY205643 | Unpublished |
| Stylommatophora | Helicina | Urocoptoidea | Urocoptidae | *Microceramus pontificus* | KY132095 | Unpublished |
| Stylommatophora | Orthurethra | - | Pupillidae | *Gastrocopta cristata* | KC185403 | Unpublished |
| Stylommatophora | Orthurethra | - | Pupillidae | *Pupilla muscorum* | KC185404 | Unpublished |
| Stylommatophora | Orthurethra | - | Vertiginidae | *Vertigo pusilla* | KC185405 | Unpublished |
| Stylommatophora | Orthurethra | Orculoidea | Orculidae | *Orcula dolium* | KJ867421 | Groenenberg et al. 2017 |
| Stylommatophora | Orthurethra | Pupilloidea | Achatinellidae | *Achatinella fulgens* | MG925058 | Price et al. 2018 |
| Stylommatophora | Orthurethra | Pupilloidea | Achatinellidae | *Achatinella mustelina* | KU525108 | Price et al. 2016a |
| Stylommatophora | Orthurethra | Pupilloidea | Achatinellidae | *Achatinella sowerbyana* | KX356680 | Price et al. 2016b |
| Stylommatophora | Orthurethra | Pupilloidea | Achatinellidae | *Partulina redfieldi* | MG925057 | Price et al. 2018 |
| Systellommatophora | - | Onchidioidea | Onchidiidae | *Onchidella celtica* | NC_012376 | Grande et al. 2008 |
| Systellommatophora | - | Onchidioidea | Onchidiidae | *Onchidium reevesii* | NC_068812 | Unpublished |
| Systellommatophora | - | Onchidioidea | Onchidiidae | *Peronia verruculata* | NC_068813 | Unpublished |
| Systellommatophora | - | Onchidioidea | Onchidiidae | *Platevindex mortoni* | OP311641 | Unpublished |
| Ellobiida | - | Ellobioidea | Ellobiidae | *Carychium tridentatum* | KT696545 | Harasewych et al. 2011 |
| Ellobiida | - | Ellobioidea | Ellobiidae | *Ellobium chinense* | NC_034292 | Jun et al. 2016 |
| Hygrophila | - | Lymnaeoidea | Lymnaeidae | *Ampullaceana lagotis* | MN175602 | Qin et al. 2019 |
| Hygrophila | - | Lymnaeoidea | Lymnaeidae | *Galba truncatula* | MT862422 | Unpublished |
| Hygrophila | - | Lymnaeoidea | Lymnaeidae | *Lymnaea stagnalis* | MW221941 | Unpublished |
| Note: Bold indicates the species sequenced in this study. | | | | | | |

References cited in this table are as follows:

Ahn S-J, Martin R, Rao S, Choi M-Y (2017) The complete mitochondrial genome of the gray garden slug *Deroceras reticulatum* (Gastropoda: Pulmonata: Stylommatophora). Mitochondrial DNA Part B 2: 255-256.

Damatac AMI, Fontanilla IKC, Center PG (2019) Complete Mitochondrial Genome and Novel Gene Organization of *Ryssota otaheitana* (Pulmonata: Chronidae), and its Implications on the Stylommatophora Phylogeny. Philippine Journal of Science 148: 167-180.

Deng P-J, Wang W-M, Huang X-C, Wu X-P, Xie G-L, Ouyang S (2016) The complete mitochondrial genome of Chinese land snail *Mastigeulota kiangsinensis* (Gastropoda: Pulmonata: Bradybaenidae). Mitochondrial DNA Part A 27: 1441-1442.

Doğan Ö, Schrödl M, Chen Z (2020) The complete mitogenome of *Arion vulgaris* Moquin-Tandon, 1855 (Gastropoda: Stylommatophora): mitochondrial genome architecture, evolution and phylogenetic considerations within Stylommatophora. PeerJ 8: e8603.

Gaitán-Espitia JD, Nespolo RF, Opazo JC (2013) The complete mitochondrial genome of the land snail *Cornu aspersum* (Helicidae: Mollusca): intra-specific divergence of protein-coding genes and phylogenetic considerations within Euthyneura. PLoS One 8: e67299.

Gonzalez VL, Kayal E, Halloran M, Shrestha Y, Harasewych M (2016) The complete mitochondrial genome of the land snail *Cerion incanum* (Gastropoda: Stylommatophora) and the phylogenetic relationships of Cerionidae within Panpulmonata. Journal of Molluscan Studies 82: 525-533.

Grande C, Templado J, Zardoya R (2008). Evolution of gastropod mitochondrial genome arrangements. BMC Evolutionary Biology 8: 1-15.

Groenenberg D, Harl J, Duijm E, Gittenberger E (2017) The complete mitogenome of *Orcula dolium* (Draparnaud, 1801); ultra-deep sequencing from a single long-range PCR using the Ion-Torrent PGM. Hereditas 154: 1-10.

Groenenberg DS, Pirovano W, Gittenberger E, Schilthuizen M (2012) The complete mitogenome of *Cylindrus obtusus* (Helicidae, Ariantinae) using Illumina next generation sequencing. BMC Genomics 13: 1-11.

Guzmán LB, Vogler RE, Beltramino AA (2021) The mitochondrial genome of the semi-slug *Omalonyx unguis* (Gastropoda: Succineidae) and the phylogenetic relationships within Stylommatophora. PLoS One 16: e0253724.

Harasewych M, González VL, Windsor AM, Halloran M (2017) The complete mitochondrial genome of *Cerion uva uva* (Gastropoda: Panpulmonata: Stylommatophora: Cerionidae). Mitochondrial DNA Part B 2: 159-160.

Harasewych M, Sikaroodi M, Gillevet PM (2011) The Delray Beach, Florida, colony of *Cerion* (*Paracerion*) *tridentatum costellata* Pilsbry, 1946 (Gastropoda: Pulmonata: Cerionidae): Evidence for indirect Cuban origins. Nautilus 125: 173-181.

Hatzoglou E, Rodakis GC, Lecanidou R (1995) Complete sequence and gene organization of the mitochondrial genome of the land snail *Albinaria coerulea*. Genetics 140: 1353-1366.

He Z-P, Dai X-B, Zhang S, Zhi T-T, Lun Z-R, Wu Z-D, Yang T-B (2016) Complete mitochondrial genome of the giant African snail, *Achatina fulica* (Mollusca: Achatinidae): a novel location of putative control regions (CR) in the mitogenome within Pulmonate species. Mitochondrial DNA Part A 27: 1084-1085.

Huang C-W, Lin S-M, Wu W-L (2016) Mitochondrial genome sequences of land snails *Aegista diversifamilia* and *Dolicheulota formosensis* (Gastropoda: Pulmonata: Stylommatophora). Mitochondrial DNA Part A 27: 2793-2795.

Hunter SS, Settles ML, New DD, Parent CE, Gerritsen AT (2016) Mitochondrial genome sequence of the Galápagos endemic land snail *Naesiotus nux*. Genome Announcements 4: e01362-01315.

Jun J, Choi EH, Kil HJ (2016) Complete mitochondrial genome of the Endangered species *Ellobium chinense* (Pulmonata, Ellobiidae) from Korea. Mitochondrial DNA Part B 1: 939-940.

Lin J-H, Zhou W-C, Ding H-L, Wang P, Ai H-M (2016) The mitochondrial genome of the land snail Cernuella virgata (Da Costa, 1778): the first complete sequence in the family Hygromiidae (Pulmonata, Stylommatophora). ZooKeys: 55.

Minton RL, Cruz MAM, Farman ML, Perez KE (2016) Two complete mitochondrial genomes from *Praticolella mexicana* Perez, 2011 (Polygyridae) and gene order evolution in Helicoidea (Mollusca, Gastropoda). ZooKeys: 137-154.

Petrusek A, Rovatsos M (2019) The complete mitogenome of *Helixpomatia* and the basal phylogeny of Helicinae (Gastropoda, Stylommatophora, Helicidae). ZooKeys 827: 19.

Price MR, Forsman ZH, Knapp I, Hadfield MG, Toonen RJ (2016a) The complete mitochondrial genome of *Achatinella mustelina* (Gastropoda: Pulmonata: Stylommatophora). Mitochondrial DNA Part B 1: 175-177.

Price MR, Forsman ZH, Knapp I, Toonen RJ, Hadfield MG (2018) A comparison of mitochondrial genomes from five species in three genera suggests polyphyly in the subfamily Achatinellinae (Gastropoda: Pulmonata: Stylommatophora: Achatinellidae). Mitochondrial DNA Part B 3: 611-612.

Price MR, Forsman ZH, Knapp IS, Toonen RJ, Hadfield MG (2016b) The complete mitochondrial genome of *Achatinella sowerbyana* (Gastropoda: Pulmonata: Stylommatophora: Achatinellidae). Mitochondrial DNA Part B 1: 666-668.

Qin D-M, Huang X-C, Yang L-M, Liu X-J, Wu R-W, Ouyang S, Wu X-P, Wang S-H (2019) Complete mitochondrial genome of the radicine pond snail *Radix plicatula* (Gastropoda: Lymnaeidae). Mitochondrial DNA Part B 4: 2861-2862.

Romero PE, Weigand AM, Pfenninger M (2016) Positive selection on panpulmonate mitogenomes provide new clues on adaptations to terrestrial life. BMC Evolutionary Biology 16: 1-13.

Terrett J, Miles S, Thomas R (1996) Complete DNA sequence of the mitochondrial genome of *Cepaea nemoralis* (Gastropoda: Pulmonata). Journal of Molecular Evolution 42: 160-168.

Wang P, Yang H-F, Zhou W-C, Hwang C-C, Zhang W-H, Qian Z-X (2014) The mitochondrial genome of the land snail *Camaena cicatricosa* (Müller, 1774) (Stylommatophora, Camaenidae): the first complete sequence in the family Camaenidae. ZooKeys: 33-48.

Wang P, Yang S-P, Lin J-H, Zhang M-Z, Zhou W-C (2018) The mitochondrial genome of the land snail *Theba pisana* (Müller, 1774) (Stylommatophora: Helicidae): The first complete sequence in the genus *Theba*. Mitochondrial DNA Part B 3: 798-800.

White TR, Conrad MM, Tseng R, Balayan S, Golding R, de Frias Martins AM, Dayrat BA (2011) Ten new complete mitochondrial genomes of pulmonates (Mollusca: Gastropoda) and their impact on phylogenetic relationships. BMC Evolutionary Biology 11: 1-15.

Yang X, Xie G-L, Wu X-P, Ouyang S (2016) The complete mitochondrial genome of Chinese land snail *Aegista aubryana* (Gastropoda: Pulmonata: Bradybaenidae). Mitochondrial DNA Part A 27: 3538-3539.
