# Supplementary material for: ﻿Complete mitochondrial genomes of the slugs Deroceraslaeve (Agriolimacidae) and Ambigolimaxvalentianus (Limacidae) provide insights into the phylogeny of Stylommatophora (Mollusca, Gastropoda)
Source: Zookeys. 2023 Jul 31;1173:43–59. doi: 10.3897/zookeys.1173.102786 (PMC10407649; doi:10.3897/zookeys.1173.102786)
Supplement: Supplementary material 3 — The secondary structures of tRNA genes inferred for the mitogenome of Ambigolimaxvalentianus [file zookeys-1173-043_article-102786__-s003.pdf]

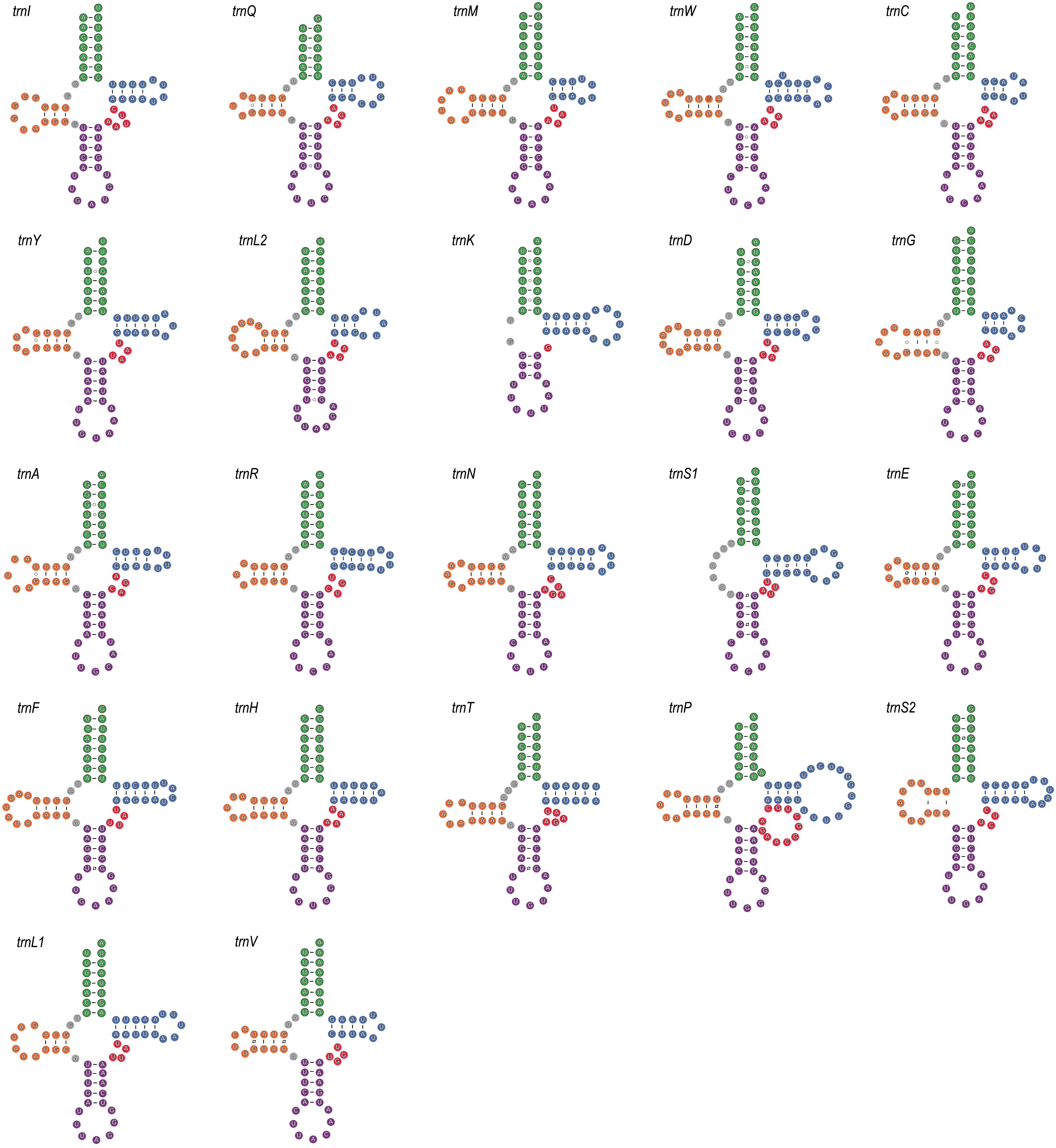

figure S2. The secondary structures of tRNA genes inferred for the mitogenome of *Ambigolimax valentianus*.
