# Supplementary figures and images for: ﻿Complete mitochondrial genomes of the slugs Deroceraslaeve (Agriolimacidae) and Ambigolimaxvalentianus (Limacidae) provide insights into the phylogeny of Stylommatophora (Mollusca, Gastropoda)
Source: Zookeys. 2023 Jul 31;1173:43–59. doi: 10.3897/zookeys.1173.102786 (PMC10407649; doi:10.3897/zookeys.1173.102786)

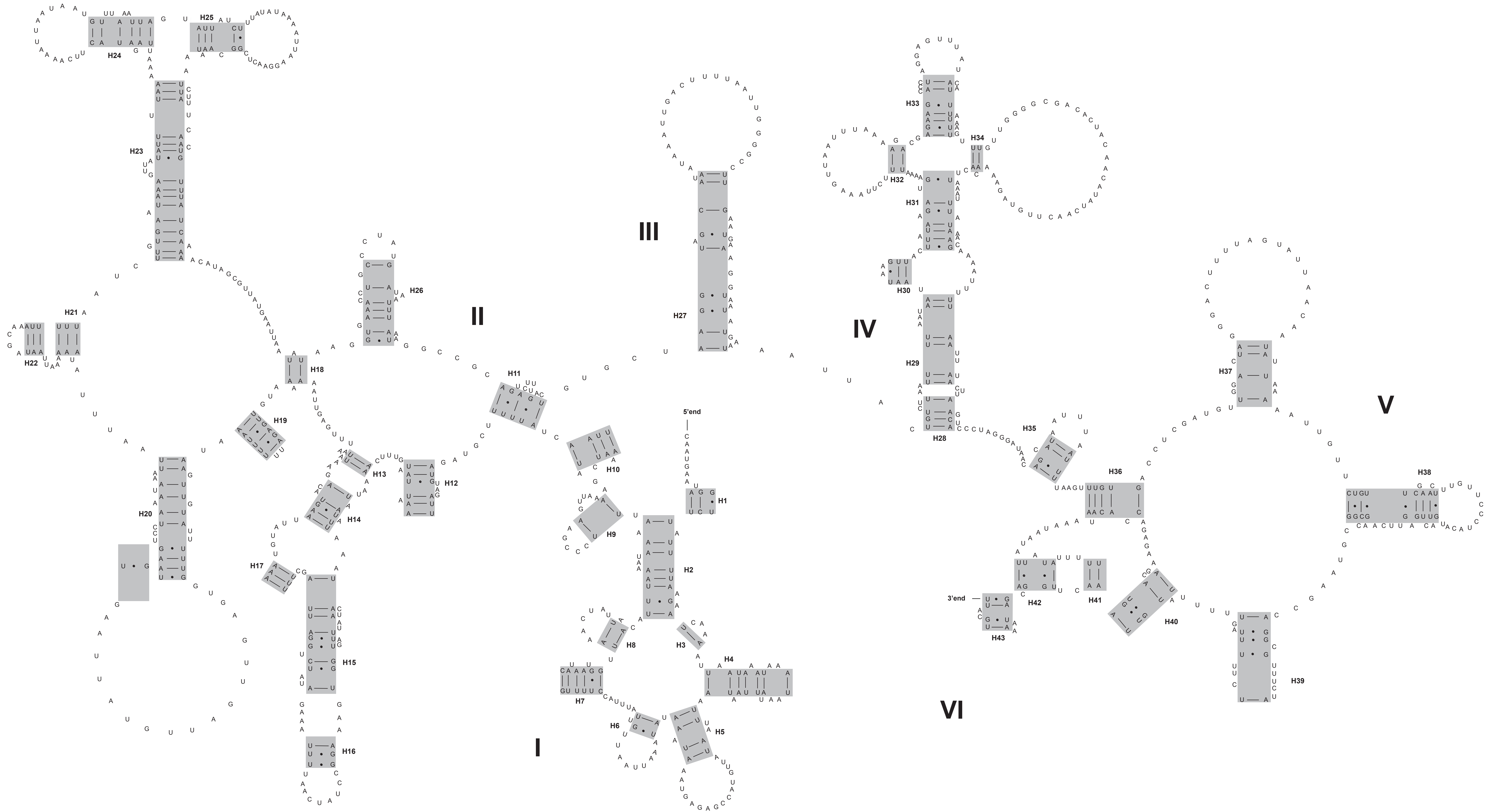

figure S3. The secondary structure of *rrnL* inferred for *Ambigolimax valentianus*.

Supplement: Supplementary material 4 — The secondary structure of rrnL inferred for Ambigolimaxvalentianus [file zookeys-1173-043_article-102786__-s004.pdf]

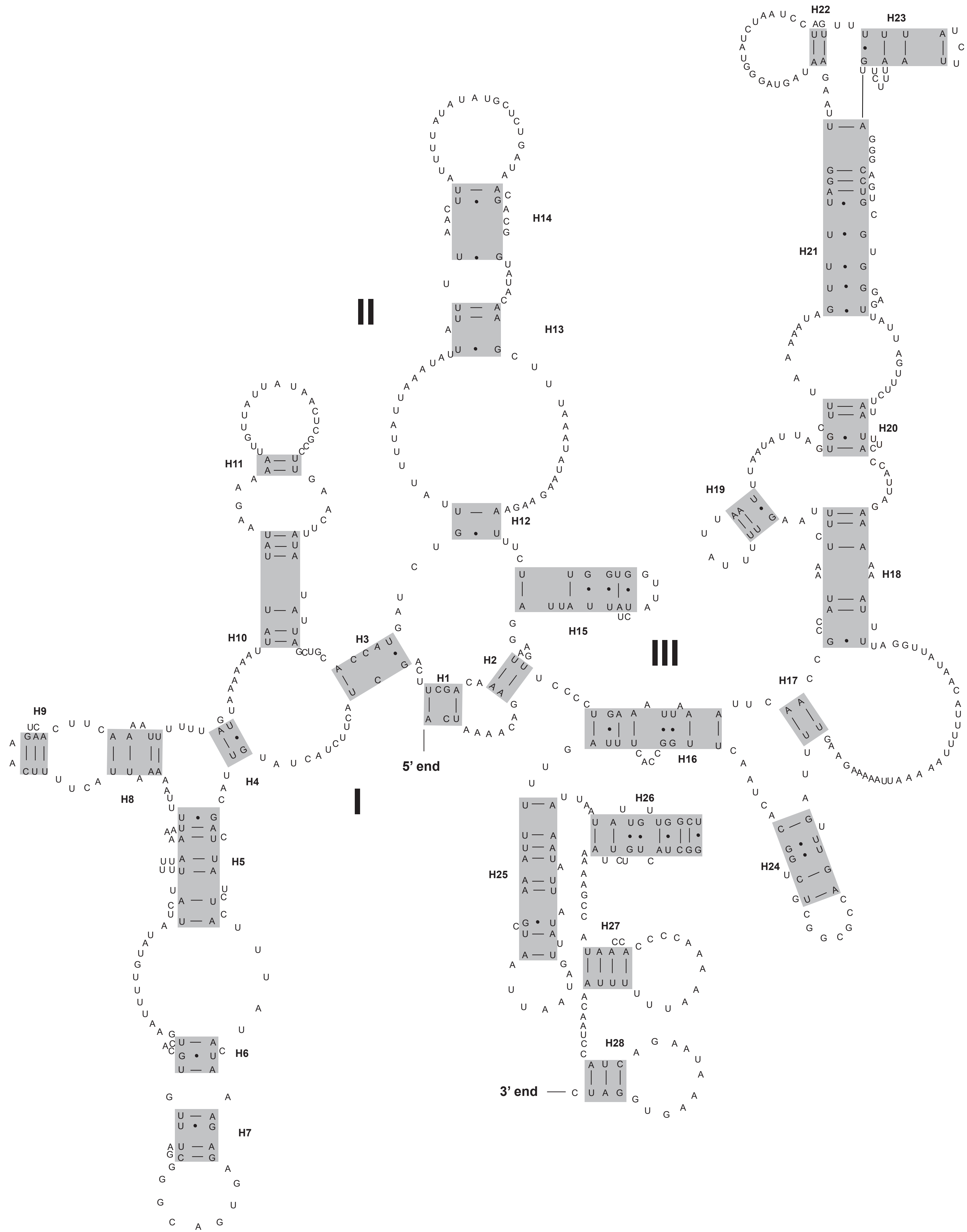

figure S4. The secondary structure of *rrnS* inferred for *Ambigolimax valentianus*.

Supplement: Supplementary material 5 — The secondary structure of rrnS inferred for Ambigolimaxvalentianus [file zookeys-1173-043_article-102786__-s005.pdf]
